# Alone But Supported: A Qualitative Study of an HIV Self-testing App in an Observational Cohort Study in South Africa

**DOI:** 10.1007/s10461-019-02516-6

**Published:** 2019-05-02

**Authors:** Ricky Janssen, Nora Engel, Aliasgar Esmail, Suzette Oelofse, Anja Krumeich, Keertan Dheda, Nitika Pant Pai

**Affiliations:** 1 Department of Health, Ethics & Society, Research School for Public Health and Primary Care, Maastricht University, Postbus 616, 6200 MD Maastricht, The Netherlands; 2 Division of Pulmonology, Department of Medicine, Centre for Lung Infection and Immunity, UCT Lung Institute, University of Cape Town, Anzio Road, Cape Town, 7925 South Africa; 3 Department of Immunology and Infection, Faculty of Infectious and Tropical Diseases, London School of Hygiene and Tropical Medicine, Keppel St, Bloomsbury, London, WC1E 7HT UK; 4 Division of Clinical Epidemiology, Department of Medicine, McGill University and Research Institute of the McGill University Health Centre, 5252 de Maisonneuve W, Montreal, H4A 3S5 Canada

**Keywords:** mHealth, Counselling, Privacy, Stigma, Test access

## Abstract

HIV self-testing has the potential to improve test access and uptake, but concerns remain regarding counselling and support during and after HIV self-testing. We investigated an oral HIV self-testing strategy together with a mobile phone/tablet application to see if and how it provided counselling and support, and how it might impact test access. This ethnographic study was nested within an ongoing observational cohort study in Cape Town, South Africa. Qualitative data was collected from study participants and study staff using 33 semi-structured interviews, one focus group discussion, and observation notes. The app provided information and guidance while also addressing privacy concerns. The flexibility and support provided by the strategy gave participants more control in choosing whom they included during testing. Accessibility concerns included smartphone access and usability issues for older and rural users. The adaptable access and support of this strategy could aid in expanding test access in South Africa.

## Resumen

El autotest del VIH puede mejorar su acceso y uso, pero, existe inquietud sobre el asesoramiento y apoyo al paciente durante y después del autotest. Investigamos una estrategia de autotest oral con una aplicación para teléfono/tableta para observar cómo proporcionaría asesoramiento y apoyo, e influenciaría el acceso al test. Este estudio etnográfico fue anidado en un estudio de cohorte observacional en curso en Ciudad del Cabo, Sudáfrica. Recogimos datos cualitativos de participantes y personal del estudio empleando 33 entrevistas semiestructuradas, una discusión de grupo focal, y apuntes de observación. La aplicación proporcionó información y orientación, abordando inquietudes sobre privacidad. La flexibilidad y apoyo al paciente proporcionaron a los participantes más control sobre quién involucraban en el test. Problemas de accesibilidad incluyeron acceso y uso de Smartphone en usuarios mayores y residentes rurales. El acceso adaptable y apoyo de esta estrategia podría ayudar a expandir el acceso al test en Sudáfrica.

## Introduction

The World Health Organization (WHO) and the Joint United Nations Programme on HIV/AIDS (UNAIDS) describe HIV testing services as a crucial entry point to other services such as HIV prevention, treatment, care and support [1]. Advantages of HIV testing include health benefits of early treatment initiation, prevention of transmission to partners and reduction of community viral load [1]. But in countries such as South Africa, which had the largest HIV epidemic in the world as of 2016 [2], key challenges to HIV testing sometimes persist in health care facilities, impeding pace of progress. These include stigma and discrimination [3, 4], perceived lack of privacy and confidentiality from healthcare workers [3, 5], and long clinic wait times [3].

The WHO has recommended HIV self-testing (HIVST) as an alternative to conventional facility based testing [1]. HIVST is a process in which a person, often in private, collects his or her own specimen (oral fluid or blood) and performs and interprets their test [6]. HIVST has been demonstrated to positively impact HIV test uptake and frequency [7] and could lead to early diagnosis and treatment [8] while overcoming confidentiality concerns [5, 9, 10]. Clinic staff and HIV counselors in South Africa have to perform a high number of tests each day [11]. Allowing patients to test on their own could reduce the workload and stress on staff and allow patients to avoid long queues at the clinic.

Yet, concerns have been raised regarding the lack of counselling and support during and after the self-testing process [8]. Research has shown that some participants struggle to complete oral self-testing on their own due to unclear instructions [12]. One study showed that only 39% of people correctly performed all steps of an oral HIV self-test when doing the test alone with written instructions [12], whereas another study showed that user error rate was negligible when completing an oral HIV self-test with the help of a counsellor [13]. In addition, many testers desire in-person support during the test process [9] or need encouragement and reassurance from those providing pre-test counselling in order to proceed with testing [4]. The current data around HIVST is growing, but still inadequate from sub-Saharan African countries when considering the burden and distribution of HIV, and research regarding appropriate counselling services and implementation of HIVST is needed [14]. In addition, previous qualitative research suggests further exploration of counselling provision during HIVST is necessary, specifically within South Africa [15]. Currently, testers only have the option to choose between the privacy of HIVST with limited counselling and support, or seeking the option of face-to-face counselling provided in conventional clinic-based testing that comes with concerns of privacy and stigmatization. The findings presented in this paper highlight the importance of identifying and evaluating HIVST strategies which attempt to go beyond this dichotomy in testing options by showing how more flexible strategies can help to address issues with the current offer of self-testing.

Mobile health (mHealth) strategies have been suggested as one of the additional services accompanying HIVST to ensure adequate support and counselling [8] thereby allowing for privacy and support simultaneously. mHealth strategies, which are described as mobile communications technology used to deliver health care [16], can help surmount issues related to logistics, culture and economics for groups that are either medically or socially marginalized [17]. In the context of HIV care, mHealth strategies have been effective in increasing clinic attendance, treatment adherence and turnaround time from testing to treatment [18]. Furthermore, strategies which utilize mobile technologies for HIV self-testing have been shown to support linkage to care [19, 20].

Although there are expectations around how a technology will work, in practice, technologies only work in relation to people and other technologies [21]. Therefore, studying the interactions between new technologies and their users is necessary to understand if a technology is working as intended [21]. Previous research on HIVST raises concerns about support while doing the test and post-test counselling, but there is little research on HIVST strategies which aim to address these concerns. The purpose of this research was to explore how people implemented, used and experienced a new mHealth-based HIVST strategy. The present study used an ethnographic approach, which involved spending time with the people using and implementing the app-based HIVST strategy, observing their practices and experiences, and describing the world from their standpoint [22]. This qualitative study was nested within an observational cohort study in Cape Town, South Africa. The cohort study aimed to evaluate a new HIVST strategy which uses a confidential smartphone application (app) (for android and IOS) to help users complete an oral HIVST strategy while also linking them to counselling and care. This paper is unique in that, to the best of our knowledge, there is currently no qualitative research available that has examined the interaction between a smartphone-based HIVST app, in a supervised and unsupervised HIVST context, and HIV self-testers in South Africa. It is unclear how users will interact with the app-based HIVST. Using qualitative methods, this study aimed to; 1) investigate if and how the HIVST app strategy plays a role in allowing users to combine counselling, support and privacy during the testing process and 2) investigate how a smartphone-based app strategy might impact users’ ability to access HIV testing.

## Methods

Qualitative data was collected between February and March 2017 from three primary health care facilities located in the township areas of Cape Town, South Africa where the observational cohort study was being conducted. Two HIVST strategies were being offered: (a) unsupervised HIVST and (b) supervised HIVST. If participants had a smartphone and the app downloaded to their phone, they could choose to do the unsupervised testing strategy at home. Another unsupervised option was also observed: if the participant did not have a phone, had issues downloading the app or had issues accessing Wi-Fi, or did not want to test at home, they could still choose to perform “unsupervised HIVST” in a kiosk set up at a clinic on a tablet provided by the study, but without assistance from study counsellors. In the supervised HIVST option, participants chose to complete the testing and app using a tablet provided by the study, while under the direct supervision of study staff at the clinic. The HIVST at the clinics was arranged in a small building behind the outpatient clinics where the study was being conducted (for this paper, this location is referred to in general as “the clinic”).

### Study Design, Data Collection and Participants

This study included interviews with study participants and a focus group discussion (FGD) and interviews with study staff from the observational cohort study described above. We conducted 30 semi-structured interviews with participants enrolled in the observational cohort study. Participants were eligible for an interview if they had completed self-testing using the app, were able to come to the clinic, and felt comfortable with doing an interview. Participants were interviewed in a private room in the clinic, or in a private room in the building behind the clinic, after testing. Study participants were purposively sampled for interviews with the aim of equally representing gender, age, the experimental groups (unsupervised and supervised testing) and the three study sites (please see Table 1). We also aimed to ensure those participants who tested positive and those who tested negative were represented within the sample.

**Table 1:** Characteristics of study participants (self-testers) who were interviewed

	Female (F)	Male (M)
Total (N)	16	14
Mean age (SD)	26.6 (7.1)	26.4 (5.5)
Clinic site		
Clinic 1	4	6
Clinic 2	6	4
Clinic 3	6	4
Testing method		
Supervised (S)	7	4
Unsupervised at home (UH)	3	6
Unsupervised at clinic (UC)	6	4
HIV status^a^		
HIV negative	11	12
HIV positive	5	2

^a^Based on reported test result

We also conducted one FGD with study staff (n = 6) comprising of nurses and health care workers from each of the three study sites. In addition, we conducted two interviews with nurses and one joint interview with two medical officers from the study who could not attend the FGD. For an explanation of the participant code descriptions used in the results section, please see Table 2. Non-participant observation was carried out at clinics, and a mix of non-participant and participant observation occurred during team meetings and in the study team’s office.

**Table 2:** Participant code description

Type of participant	Participant #	Code	Participant characteristics
Study participants (self-testers)^a^	1–30		
		UH	Unsupervised tester at home
		UC	Unsupervised tester at the clinic
		S	Supervised tester
		M	Male participant
		F	Female participant
Staff participants^b^			
	1–3	HFGD	Health care worker in focus group discussion
	1–3	NFGD	Nurse in focus group discussion
	1–2^c^	NI	Nurse in interview
	1–2	MO	Medical officer in interview

^a^E.g. #1, UHF would mean that the first study participant interviewed was a female who did unsupervised self-testing at home

^b^E.g. #1, HFGD would be the health care worker designated number 1 in the Focus Group Discussion

^c^The nurses interviewed are different nurses than in the FGD

A topic guide was used during the interviews and FGD with study staff and study participants to improve consistency of data collection and to ensure that relevant themes were covered in a flexible manner [23]. The topic guide used for interviews with the study participants was designed to explore; reasons for HIV testing, previous testing experiences, reasons for choosing the HIVST option, experiences and process of using the self-test/app, trusting the testing method, smartphone and internet access, potential cost of the ST strategy, and where people might access HIVST and app in the future. The topic guide for the interviews and FGD with study staff covered topics including; challenges of the HIVST strategy, possible solutions, experiences with the app, impact on the HIV continuum of care (i.e. diagnosis, treatment initiation, and adherence) and differences between HIVST and conventional HIV testing.

The interviews and FGD were conducted in English by the first author. In some instances, someone was present to translate into English during the interview. All interviews were digitally recorded with the consent of the participant.

### Data Analysis

Audio files and notes were transcribed. Data analysis was done using NVIVO 9 (QSR International). A coding list was compiled in close coordination between researchers [RJ and NE] using literature, research objectives and themes identified while transcribing the data. The study used an ethnographic approach which allowed data collection and analysis to be done in a cyclical manner [22]. Results were emergent and relevant issues were arrived at in an inductive way [22]. The coding list was tested on a few interviews and refined, and the data was then organized by grouping using these codes [24]. During analysis, themes identified from coding the overall data were then used to characterize individual experiences and practices of participants [24].

### Ethical Clearance

The study was approved by Institutional Review Boards of two institutions including; the McGill University Health Centre, Montreal, Canada and the University of Cape Town, Cape Town, South Africa.

## Results

The results present themes identified in experiences and practices of those interacting with the HIVST app strategy. Themes include: counselling and support in relation to the app, space and privacy in relation to the self-testing (ST) strategy, and negotiation of different levels of support which were sought and provided in different ways during ST.

### The App Providing Support

The app supported participants in doing the test, interpreting ST results, contextualizing these results via provision of information on HIV, personal risk of acquiring HIV, and by giving advice on what to do after they received a result. Participants generally perceived the instructions in the app as simple and straightforward and they helped first time oral testers to familiarize themselves with the technique (#6, UCF) and build confidence to test on their own. One participant illustrated this point while referring to the app’s instructional video;

“Yes I did trust myself [in doing the test] because I saw there by the video how you must do step by step how to test yourself. So, it wasn’t that difficult, it was easy.” (#24, UHM)

In addition to the instructional video, the app included a lot of information about HIV (#14, SF; #1 HFGD; #3, NFGD). One participant compared the app to the role of the counsellor saying; “The app is already counselling you, you don’t need a counsellor to counsel you again. You just go in and do the test.” (#1, UHF). Another participant said that doing the app was like talking to a person, but with the added benefit of not having to answer sensitive risk behavior questions with an actual person (#30, SF). Instead, the app asked sensitive questions about condom use, number of sexual partners, alcohol and drug use prior to sexual interactions etc., and thereby assessed one’s risk for HIV.

After completing the test, the app then took participants through the process of interpreting the result. The app told participants if they were HIV negative or positive when they clicked the picture on the app that corresponded with the test result seen on the oral test (#13, SM; #1, HFGD). After receiving the results, the app stored the results and provided post-test counselling on what to do if positive (#10, SF; #3, SM; #3 NFGD), or negative (#25, UCF; #3, SM; #3 NFGD). For linkages, it showed a map with the location of a few clinics offering counselling and treatment and support. Contact information for the nurses was also provided. One participant gave an example of how the app supported and reassured her after receiving a positive result saying;

“Uh I read, I get a message [on the app] is explaining how to do when you have HIV. I get a message when you’re positive, what supposed to do and how to treat and it said don’t be stressed about your results. Go to the clinic and get to counsel classes and figure it.” (#10, SF)

These examples illustrate how the app provided information and counselling without the presence of clinic staff. The support provided through the app enabled users to do testing on their own and allowed them to better negotiate their privacy.

### Negotiating Space

In order to achieve the privacy promised by the HIVST strategy, participants who chose to test at home first needed to negotiate a private space at home to conduct the test. According to participants who chose for unsupervised self-testing at home, they were able to access a private space. However, testers did take some precautions to ensure their privacy. One participant asked her cousin to leave the room while she did the test. When asked why she did this, the translator said;

“She didn’t trust her because it’s her cousin not her sister, because sometimes when you’ve got quarrels then she will take you, she will spread things to others, that’s why she tell her that she must go out.” (Translator for #16, UHF)

This highlights a potential concern regarding privacy during testing at home and is a reminder that testing at home does not automatically ensure privacy. In the example mentioned here, the tester was able to negotiate privacy during the test. However, although this is a concern specific to the township population and is not applicable to all contexts, study staff mentioned that many homes in the region were crowded and that this could impede on a tester’s ability to test privately (#1, #2, NI). However, the option to do unsupervised HIVST at the clinic offered a way out. A participant highlighted this point;

“But either way, I don’t know if I would have done it at home eh? ‘Cause I’m, with the dynamics at home, we’re like um too many people in one space and so privacy is a bit of a, so people would be like, what is that? What, what are you doing? So, it would have been tricky for me to do it at home. So that’s why I’m grateful that I actually did it here [at the clinic]. But I did it on my own.” (#27, UCF)

The flexibility of the testing process implemented during the study allowed participants to choose to test on their own at home or in a private clinic space. Next, we investigate how privacy and support were flexible and how support was sought in different ways.

### Negotiating the Level of Support

Unlike in conventional testing, study participants had more control in stipulating who and how they involved others in their self-testing process. The study allowed for varying levels of support based on the self-testing option chosen by the participant. Within these varying options, participants chose to include (or not include) others in different ways. Some participants specifically chose for the involvement of friends, partners, and clinic staff during the testing process, while others chose to test alone. For instance, one participant who tested at home had his girlfriend do the test with him, so she could help him with the instructions (#28, UCM). Another participant who did the self-test at home had a friend help him do the test and read the results (#18, UHM). When asked why he chose to have his friend present, he highlighted; “Because he’s the one who’s more close to me, I always tell him my problems and all that stuff. I always share my problems with him.” (#18, UHM). This highlights that some testers will seek additional support and that, although they decide to test at home, does not necessarily mean they choose to test alone. In contrast, there were participants who wanted to do the testing completely alone. One participant highlighted this when asked why he would rather test at home saying;

“Because you see, (…) I don’t like testing myself in front of other people. You see? It’s right on my own, it’s because I’m at my own space. I would know my own status, how it is, not anyone checking behind me if okay, he’s negative or positive. (…)” (#24, UHM).

However, another participant brought up the concern that not every HIV tester will have a support system at home and that people may want to test at the clinic because they want a health professional to contextualize their test result and tell them that they will be okay;

“We would rather do it at the clinic ‘cause you know at the clinic there is a doctor who will tell you what to do, you what you want to hear in fact.” (#1, UHF)

When asked what the participant meant by “what you want to hear” they responded; “That it will be alright, that you will be fine. There is nothing wrong, this is not the end of the world. People who will tell you that.” (#1, UHF). This shows that some participants may want the support and reassurance of health professionals during the testing process. One nurse recalled one instance where a participant had specifically asked to do the self-testing at the clinic because the participant was too afraid to do the testing at home alone (#1, NI). Due to the app and study, testers could still do the test under supervision of clinic staff. A participant illustrated this saying;

“Um, she [the nurse] was beside me, but I, it’s the one [the testing option] that I can see on the app and the video is the one that would tell me what to do, then I would do it and it’s quite easy. She didn’t have to, you know, talk to me and I just watched the video and then I can do it.” (#8, SF)

This shows that, because of the app, a tester still had the ability to the test on their own, while they also had clinic staff near them for additional support if necessary. These examples highlight the differing levels of support that were sought by testers, and shows how the different self-testing strategies together with the support of the app allowed testers to negotiate who and how they involved others in the testing process.

### The Ability to Negotiate the Testing Process

The ability of a user to negotiate their needs in their testing process, for example the level of support and privacy, is contingent upon their ability to access or utilize the testing options available. Participants highlighted multiple factors that could influence the accessibility or usability of the app strategy.

Some participants wouldn’t bring their phone to the clinic, which was necessary to download the app, because they were afraid of being robbed (#1, NI). There were also instances where participants did not have phones, sometimes because their phone had been stolen. However, in the present study, participants were able to access a tablet at the clinic and some participants were able to borrow phones from other people.

Other concerns around whether participants would be able to use the app centered on age, demographics and language. A few staff mentioned that older people may struggle more using this technology and that this HIV-ST strategy would favor the youth demographic (#1, NI; #3, HFGD). It was also suggested that the feasibility of implementing this kind of strategy in a rural area would need to be further evaluated (#1, MO). Although language was suggested as a potential issue in using the app (#1, NI), language did not appear to be a problem according to participants. Although a Xhosa version was included in the app to reduce potential language barriers with the local community, the English version was often used instead. Participants noted that they chose the English app version instead of the Xhosa version, often at the direction of the nurses or HCW’s, because English explained things more directly.

This shows how important it is to evaluate user preferences and technology access as part of evaluating app based testing strategies as these can impact the ability of users to negotiate different aspects of the testing process.

## Discussion

In the South African context, a lack of trust in healthcare workers [3, 5], fear of discrimination on HIV status [3, 5], and long wait times at the clinic are barriers to conventional HIV testing [3]. HIVST presents a potential way of circumventing these issues by allowing people to test alone at home, but concerns remain regarding the provision of adequate pre- and post-test counselling, linkage to care, and proper test conduct [5, 12]. Furthermore, with South Africa’s 2016 policy move introducing a “treatment for all approach” [25], improvements in the overall efficiency and quality of HIV care services is crucial to its sustainability [26].

This study examined a smartphone app that is used together with an oral HIVST. The app helped people through the process of self-testing by providing counselling and care and simplifying the process of self-testing. This study demonstrates that the app addresses multiple common HIV testing barriers, such as lack of confidentiality, wait times and where to test, because it provides testing services outside of the clinic context or within the clinic but with an additional layer of privacy. Participants in this study had the option to use the app-based HIVST strategy unsupervised at home, unsupervised alone at the Kiosk around the clinic, or supervised under direct supervision of staff at the clinic. Our analysis reveals how the app strategy goes beyond the dichotomy of testing by giving testers the option of being alone while still supported during the testing process. Our analysis also reveals that the app addresses privacy issues by allowing testers to answer personal risk questions in private. Nonjudgmental disclosure has been considered a therapeutic benefit of mHealth strategies for HIV self-monitoring [27].

Our study identifies how participants chose for varying levels of support during their testing process. In a study previously conducted in Malawi and Zimbabwe, some patients preferred to have clinic staff and support available during self-testing despite confidentiality concerns [9]. However, it also highlighted that some participants did not want study staff present for the entire testing process. Technology may be used differently in practice compared to expectations [21], and this seems to be apparent in the way self-testing is used by some participants. Avoiding healthcare facilities and personnel is part of the allure of the ST strategy, but in reality, the participant’s preferences and needs during the testing process do not appear to be as straightforward. Participants may not necessarily want to avoid interaction with healthcare workers altogether, but rather, want the ability to choose when this interaction occurs. Our qualitative results highlight how the app was not only used by the participant to test alone, but was also used to better negotiate where, when, and with whom HIVST occurred. This emphasizes the nuances in the self-testing process and the ongoing tension between patients wanting privacy while also wanting support in others forms, for example from family, partners, counsellors, and providers (clinic staff). The differing needs of patients and these testing nuances also support the notion that self-testing should be regarded as a “middle road” to improve community engagement with testing, and not as a substitute for conventional HIV testing [28].

When scaling up an innovation, one needs to address the central tension between making a technology which is standardized and widely applicable, yet locally aligned [29]. Depending on how the strategy is implemented in different settings, affordability may also play a role, but further assessment is necessary to establish what costs would be acceptable to users based on the benefits provided by this strategy.

Limitations of this study include the potential lack of applicability of these results to rural test settings as this study was based in an urban/peri-urban setting where clinic services and technology access may differ. Therefore, future assessment of this strategy will need to evaluate how factors such as rural versus urban environments would impact scale up of this approach. Furthermore, our study population only recruited adult participants, and therefore further evaluation is required in the adolescent population. In addition, since our study participants consisted of those who had consented to do HIV testing as part of the wider cohort study, our results do not reflect the opinions of those who might have been too afraid to come to the clinic or seek HIV services in the first place.

## Conclusion

HIVST has the potential to increase self-test uptake, but ensuring the provision of proper support and linkage to care during the HIVST process is key for this testing approach to be widely successful. The current dichotomy in testing options forces people to choose between testing privately at home with minimal support, or through conventional testing offered in clinics, where people face concerns around lack of privacy and stigmatization. This study illustrates how an app-based HIVST strategy provided counselling and support while also enabling privacy per patient choice and preference, which addresses previous questions around how counselling options can be tailored for different users and settings [30]. The app-based strategy provided the necessary support to conduct the test in the privacy of one’s home or alone at the kiosk in clinics; yet, this study also shows that some testers chose to involve others, such as friends, family or clinic staff, in their testing process. Thus, the study shows that testers may enact the concept of self-testing in many ways and negotiate different levels of support. Finally, the flexibility of the app-based self-testing strategy in regards to privacy and support could in future enable optimization of test access and completion in the South African and beyond.
